# Managing African Swine Fever: Assessing the Potential of Camera Traps in Monitoring Wild Boar Occupancy Trends in Infected and Non-infected Zones, Using Spatio-Temporal Statistical Models

**DOI:** 10.3389/fvets.2021.726117

**Published:** 2021-10-12

**Authors:** Martijn Bollen, Thomas Neyens, Maxime Fajgenblat, Valérie De Waele, Alain Licoppe, Benoît Manet, Jim Casaer, Natalie Beenaerts

**Affiliations:** ^1^Centre for Environmental Sciences, UHasselt – Hasselt University, Hasselt, Belgium; ^2^Data Science Institute, UHasselt – Hasselt University, Hasselt, Belgium; ^3^Research Institute Nature and Forest, Brussels, Belgium; ^4^Laboratory of Aquatic Ecology, Evolution and Conservation, KU Leuven – Leuven University, Leuven, Belgium; ^5^Department of Natural and Agricultural Environment Studies, Public Service of Wallonia, Gembloux, Belgium

**Keywords:** African swine fever, camera traps, occupancy, spatio-temporal, Bayesian inference, Stan

## Abstract

The recent spreading of African swine fever (ASF) over the Eurasian continent has been acknowledged as a serious economic threat for the pork industry. Consequently, an extensive body of research focuses on the epidemiology and control of ASF. Nevertheless, little information is available on the combined effect of ASF and ASF-related control measures on wild boar (*Sus scrofa*) population abundances. This is crucial information given the role of the remaining wild boar that act as an important reservoir of the disease. Given the high potential of camera traps as a non-invasive method for ungulate trend estimation, we assess the effectiveness of ASF control measures using a camera trap network. In this study, we focus on a major ASF outbreak in 2018–2020 in the South of Belgium. This outbreak elicited a strong management response, both in terms of fencing off a large infected zone as well as an intensive culling regime. We apply a Bayesian multi-season site-occupancy model to wild boar detection/non-detection data. Our results show that (1) occupancy rates at the onset of our monitoring period reflect the ASF infection status; (2) ASF-induced mortality and culling efforts jointly lead to decreased occupancy over time; and (3) the estimated mean total extinction rate ranges between 22.44 and 91.35%, depending on the ASF infection status. Together, these results confirm the effectiveness of ASF control measures implemented in Wallonia (Belgium), which has regained its disease-free status in December 2020, as well as the usefulness of a camera trap network to monitor these effects.

## Introduction

African swine fever (ASF), a viral disease that causes high mortality among domestic pigs (*Sus scrofa domesticus*) and wild boar (*Sus scrofa*), originates from East Africa and is regarded as one of the most important threats to the European pig industry. Recently, ASF has been re-introduced to the wild boar populations on the European mainland, presumably due to infected meat spills in the environment (1). Most likely, this spillage mediated the recent spread of ASF through a new epidemiological cycle, designated the wild boar-habitat cycle, which involves both direct and indirect viral transmissions. Direct transmissions occur through contacts among wild boar, whereas indirect cases result from viral reservoirs in the environment, such as ASF-infected carcasses (2). This new role of wild boar in the epidemiology of ASF has led to new management guidelines of wild boar populations in infected areas (3, 4). Management strategies include continuous carcass removal from the infected zone, coupled with intense culling of wild boar within a buffer zone (5). Together, these strategies are expected to effectively reduce ASF transmission by removing viral sources from the environment in the infected zone and by depleting the susceptible wild boar population in the buffer zone. The latter is essential, since the number of individuals remaining in the host population of the buffer zone will determine the probability of the spread to a non-infected area, i.e., host threshold density. In the infected zone, after the epidemic phase, culling of the remaining wild boar will determine the probability for the disease to become endemic, i.e., critical community size (6). To evaluate measures aimed at counteracting ASF, sound information on the joint effect of the disease and culling efforts on population trends of wild boar within the managed areas is crucial (3).

Over the last decade, the use of remote cameras, henceforth referred to as camera traps (CTs), has become popular when monitoring trends in medium-size to large-size mammals, including wild boar (7, 8). Photographic captures (i.e., detections) by CTs can be translated into information on the distribution and density of a focal species. However, density estimation by CTs is still hindered by imperfect detection in many cases (i.e., not detecting a focal species, when present) (9). Given the elusiveness and nocturnality of wild boar, it is among the species subjected to severely limited detectability. Moreover, traditional density estimation methodology requires individual identification, hence cannot be applied to many common species that lack natural markings, including wild boar (10, 11). Statistical frameworks such as the random encounter model (REM) allow for density estimation of unmarked populations, while accounting for imperfect detection, using CTs (12, 13). However, the need for auxiliary data collection restricts the use of REM in many cases (14). Occupancy models on the other hand overcome both imperfect detectability and the need for individual recognition of animals, without requiring additional information. They proceed by simultaneously estimating site-occupancy and the probability of detecting a focal species, given its presence (15). Extending occupancy models to so-called multi-season site-occupancy (MSO) models, enables estimating rates expressing population changes through time. One of these rates, the extinction rate, is of prime interest when assessing the combined effect of a viral disease, such as ASF, and culling efforts on a host population.

In the current study we evaluate wild boar population trends throughout the recent ASF epidemic in Wallonia (Belgium), using a camera trap network. The first cases of this outbreak were reported on September 13, 2018 (16). Further, we aim to uncover the roles of ASF and control measures (i.e., primarily culling efforts) on inferred population dynamics. To our knowledge, only one study so far attempted to quantify the effects of ASF on a wild boar population using CTs (17). Here, we develop a different statistical framework, that can be adapted to model management strategies of multiple species, in diverse settings. We apply it in our study of wild boar population dynamics in an ASF-infected and non-infected zone. The latter has been subjected to an intensive culling regime (January 2019–March 2021) during the entire ASF episode. While in the infected zone, both ASF-induced mortality and culling efforts determine wild boar extinction rates. Moreover, this zone is fenced off from the surrounding non-infected zones. Using detection/non-detection data (March 2019–May 2020) from 92 CTs, we estimate monthly site-occupancy of wild boar in both ASF-infected and non-infected zones, through a Bayesian MSO. In addition, we provide wild boar distribution maps for the area under study. Finally, we use these results to draw conclusions about the ASF management in Wallonia (Belgium). We believe this case is important beyond its own setting for two reasons: (i) wildlife managers are often asked by funders to justify the use of CT networks, hence being able to show the usefulness of CTs to monitor population trends is important; (ii) authorities need to assess the effectiveness of control measures taken to prevent the spreading of ASF. Camera trapping is an understudied method that could provide valuable information on the effectiveness of these measures.

## Materials and Methods

### Study Area

**Figure 1:**
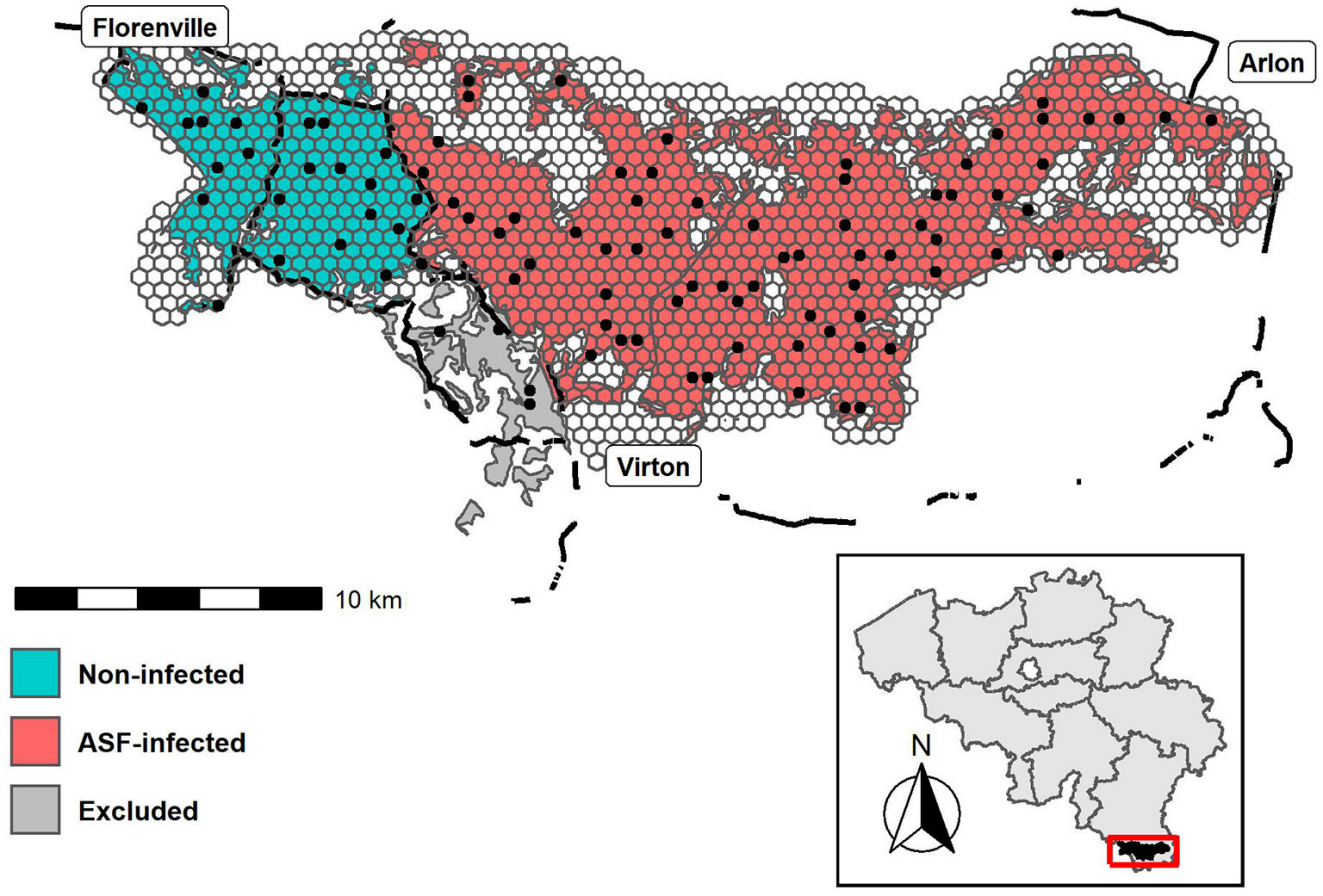
Map of the study area with the overlaying hexagonal grids. Camera deployments are indicated by the black dots. Black lines represent fences. Colors represent the African swine fever management zones; ASF-infected (red), non-infected (turquoise) and excluded (gray). The inset map (bottom) shows the study area within Belgium.

The study area (longitudes: 5.2847°E - 5.8156°E; latitudes: 49.5485°N - 49.6955°N) is situated between the cities of Virton (South), Florenville (Northwest) and Arlon (Northeast), and has been subdivided into three management zones. An ASF-infected zone (red), a non-infected zone, i.e., free of ASF (turquoise) and a zone that was excluded from the study due to its ambiguous disease status and limited number of deployments (gray), see Figure 1. It has a total forested area of 223 km^2^, part of a larger ASF management zone of 1,106 km^2^ encompassing a total of 572 forested km^2^. The study area has a cool temperate and moist climate, with a mean annual temperature of 8.94°C and 966 mm rainfall (18). The landscape is characterized by rugged terrain crossed by numerous rivers and fragmented by several roads, while its vegetation is dominated by deciduous forests.

### Camera Trap Network and Data

Within the study area, a CT network was deployed since March 2019. The network consists out of 97 Snapshot Extra Black 12.0 l HD (Dörr) cameras. For more detailed information on the camera specifications, consult Supplementary Table 1.

Camera placement was done according to a stratified random sampling scheme. Proportional to its area, a number of cameras was deployed in each management zone (stratum) (Supplementary Table 2). *A posteriori*, the strata were superimposed by a hexagonal grid layer (x-spacing of 500 m, area of 21.65 ha/ site) ensuring that each camera was assigned a unique grid cell (Figure 1). Throughout the sampling period, camera locations were fixed. All cameras were installed by mounting them on trees ~50 centimeters above ground, facing North. We did not use baiting, nor did we select for trails. Monthly check-ups were performed to determine battery levels and to verify camera operability. Each camera trigger was followed by a series of five photographs, without a delay between consecutive triggers. All images were manually annotated, using the Agouti software platform (19). After omitting data from the excluded zone (5 deployments; 5.15%) (Figure 1), we retained data from 92 deployments between March 2019 and May 2020, resulting in a total of 42,136 24-h observation periods.

We considered three classes of covariates, potentially important to explain wild boar population trends: (1) time, (2) land use, and (3) infection status. As will be clarified in section Statistical Model, for time, we evaluated three alternative definitions: (1.i) month since the start of the monitoring program (observation month; *t*), (1.ii) a binary variable indicating whether the observation month is in April–September (biannual; *BIA*_*t*_), and (1.iii) a similar indicator variable consisting of four seasonal periods, i.e., Spring, Summer, Autumn and Winter (quarterly; *QRT*_*t*_). For land use, we only considered a single covariate: the (*z*-scored) proportion of broad-leaved forest land cover class (*BL*_*t*_), which was extracted from the LifeWatch Ecotope dataset (18). Finally, infection status (*ASF*_*i*_) is encoded as follows: 1 when a site is within the ASF-infected zone, 0 otherwise (Figure 1). Note that the infection status for each zone was assigned, based on the occurrence of ASF virus (ASFV)-positive carcasses within a zone. We refer readers wanting to access the ASFV occurrence data or searching for more information on the dispersal history and dynamics of the ASFV in Belgium to Dellicour et al. (20). An overview of all covariates discussed in this section and *a priori* defined models are given in Table 1. For model-specific predictions [P (*Model*)] consult Supplementary Table 3.

**Table 1:** *A priori* defined occupancy (step 1) and detection (step 2) models.

**Model**	**Logit**(**ψ_it_**)****	**Logit**(***p*_*t*_**)****
**Occupancy models (step 1)**		
ψ1	$\alpha^{\psi }+f_{2}^{\psi }\left(x_{1}\left(i\right),\;x_{2}\left(i\right)\right)$	α^*p*^
ψ2	$\alpha^{\psi }+ASF_{i}\cdot \beta_{ASF}^{\psi }+f_{2}^{\psi }\left(x_{1}\left(i\right),\;x_{2}\left(i\right)\right)$	
ψ3	$\alpha^{\psi }+ASF_{i}\cdot \beta_{ASF}^{\psi }+BL_{i}\cdot \beta_{BL}^{\psi }+f_{2}^{\psi }\left(x_{1}\left(i\right),\;x_{2}\left(i\right)\right)$	
ψ4	$\alpha^{\psi }+ASF_{i}\cdot \beta_{ASF}^{\psi }+BL_{i}\cdot \beta_{BL}^{\psi }+t\cdot \beta_{t}^{\psi }+f_{2}^{\psi }\left(x_{1}\left(i\right),\;x_{2}\left(i\right)\right)$	
**ψ** **5**	$\alpha^{\psi }+ASF_{i}\cdot \beta_{ASF}^{\psi }+BL_{i}\cdot \beta_{BL}^{\psi }+t\cdot \beta_{t}^{\psi }+(ASF_{i}\cdot t)\cdot \beta_{ASF\cdot t}^{\psi }+f_{2}^{\psi }(x_{1}(i),\;x_{2}(i))$	
ψ(6)	$\alpha^{\psi }+ASF_{i}\cdot \beta_{ASF}^{\psi }+BL_{i}\cdot \beta_{BL}^{\psi }+f_{1}^{\psi }\left(t\right)+f_{2}^{\psi }\left(x_{1}\left(i\right),\;x_{2}\left(i\right)\right)$	
**Detection models (step 2)**		
*p*1	Top-ranking ψ – model from step 1	α^*p*^
*p*2		$\alpha^{p}+BIA_{t}\cdot \beta_{BIA}^{p}$
*p*3		$\alpha^{p}+QRT_{t}\cdot \beta_{QRT}^{p}$
**p4**		$\alpha^{p}+f_{1}^{p}\left(t\right)$

*Top-ranked models for each step are indicated in bold. ASF infection status (ASF_i_), z-scored proportion of broad-leaved tree land cover class (BL_i_), observation month (t), biannual (spring – summer, autumn – winter) seasons (BIA_t_), quarterly (spring, summer, autumn, winter) seasons (QRT_t_), smooth function for temporal variation f_1_(t), smooth function for spatial variation f_2_(lon, lat). Intercepts and slope parameters are given by α and β, respectively*.

### Statistical Model

We analyse the CT data using a longitudinal multi-season occupancy model (MSO), defined as a state-space model (21), to make inference on wild boar's site-occupancy. The sampling grids used are smaller than wild boar's home range, hence occupancy should be interpreted as habitat use (22). Detection histories were constructed using the R package *CamtrapR* (23). For site *i* = 1, 2, …, *N*, at survey day *j* = 1, 2, …, *J*, in observation month *t* = 1, 2, …, *T*, the detection history is 1 when wild boar were observed during a 24-h period (*y*_*ijt*_ = 1) or 0 when no boars are caught on camera that day (*y*_*ijt*_ = 0). These are assumed to follow a Bernoulli distribution, such that,


(1) $$\begin{array}{l} y_{ijt}|z_{it}\sim Bernoulli\left(z_{it}\;p_{ijt}\right), \end{array}$$

where *p*_*ijt*_ is the probability of detecting the focal species and *z*_*it*_ is the latent occupancy status (unoccupied *z*_*it*_ = 0; occupied *z*_*it*_ = 1) at site *i* during observation month *t*. Note that we do not use survey day-specific, nor site-specific covariates to model *p*_*ijt*_, hence the detection probability simplifies to *p*_*t*_. The occupancy status is modeled as,


(2) $$\begin{array}{l} z_{it}\sim Bernoulli\left(\psi_{it}\right), \end{array}$$

Where ψ_*it*_ is the occupancy probability, from now on simply referred to as “occupancy,” at site *i* during observation month *t*. Unlike dynamic MSO, we do not take probabilities of wild boar surviving or colonizing a site *i* from observation month *t* to *t*+1 into account, as the high degree of zero-inflation in our data complicates joint inference on all these processes. We define ϑ_*l*_ = {*p*_*ijt*_, ψ_*it*_}, which collects all processes that will be modeled as a function of covariates and random effects, using a logit link. A general model formulation for ϑ_*l*_, *l* = 1, 2, can be defined as


(3) $$\begin{array}{l} logit\left(\vartheta_{l}\right)\;={\;\alpha }_{l}+X_{l}\beta_{l}+u_{l}+f_{l,1}\left(t\right)+f_{l,2}\left(lon,lat\right), \end{array}$$

where α_*l*_ are intercepts, β_*l*_ are vectors of process-specific slope parameters with their corresponding covariate matrix *X*_*l*_. The term *u*_*l*_ models spatially unstructured overdispersion as a normally distributed random effect, *f*_*l*, 1_ is a smooth function modeling temporal variation for each observation month *t* and *f*_*l*, 2_ is an isotropic two-dimensional smooth function modeling spatial variation in occupancy patterns, for the longitude *lon* and latitude *lat* of each site's centroid. Both *f*_*l*, 1_and *f*_*l*, 2_ are modeled using Gaussian processes (GP), with an exponentiated quadratic covariance function. While *f*_*l*, 1_ uses an exact GP, we model *f*_*l*, 2_ by means of the Hilbert space reduced-rank Gaussian process (HSGP) approach as the number of sites in our study area is large (24, 25). This approach yields substantial speed gains when dealing with large number of sites through approximate series expansions of the GP's covariance function.

Model fitting was performed using *Stan* (via the R package *rstan*), a probabilistic programming language that enables Bayesian estimation through a dynamic Hamiltonian Monte Carlo (HMC) sampler (26). For each MCMC iteration, we also derive site-specific growth rates $\lambda_{it}\;=\;\frac{\psi_{it}}{\psi_{i\left(t-1\right)}}$, average monthly growth rates ${\bar{\lambda }}_{i}=\frac{1}{T-1}\sum_{t=2}^{T}\left(\lambda it\right)$ and total growth rates $\lambda_{Tot,\;i}=\frac{{\;\psi }_{iT}}{\psi_{i1}}$ in site-occupancy (27). We choose weakly informative *Student t*(3, 0, 5) priors for all the regression parameters {α_*l*_, β_*l*_} and a nonnegative *Student t*^+^(3, 0, 5) prior for the marginal standard deviation of the hyperparameters σ_*f*_1__ and σ_*f*_2__ of the GPs. For the scale parameters ρ_*f*_1__ and ρ_*f*_2__ of the GPs, we, respectively, used an inverse gamma *IG*(10.9, 4.00) and a generalized inverse Gaussian *GIG*(3, 12, 0.01), ensuring most prior evidence is placed on scales that can be estimated from the data (i.e., larger than the smallest difference between any pair of CT locations and smaller than the largest difference between any of these pairs).

The full model that would contain two random effects terms for each of these processes, in addition to covariates, was computationally infeasible to fit and, furthermore, does not necessarily reflect a sensible data-generating process. Hence, we consider a set of sensible reduced models based on ecologically plausible considerations, preventing multicollinearity, and computational feasibility (Table 1). Multicollinearity was avoided by including one of two covariates, when their Spearman rho correlation estimate |*r*_*s*_| < 0.6. Subsequently, we select the most appropriate model by means of a model selection procedure.

Model selection through approximate leave-one-out cross-validation was performed using the R package *loo* (28). Following the authors' recommendations, leave-one-out (LOO) expected log-predictive densities were used to rank our *a priori* selected candidate models (Table 2). Our ranking procedure consists of a two-step approach. First, the top-ranked occupancy model is retained by comparing LOO for selected combinations of fixed and random effects at the occupancy (ψ) level, while keeping detectability (*p*) constant (Table 2, step 1). Secondly, the detection process is modeled using fixed effects only, while adopting the top-ranked occupancy model from step 1 (Table 2, step 2).

**Table 2:** Model selection of candidate occupancy models (step 1) and detection models (step 2).

**Model**	**P_**D**_**	**SE(P_**D**_)**	**LOO**	**SE(LOO)**	**Δ LOO**	**SE(Δ LOO)**
**Occupancy models (step 1)**						
ψ(5)	45.55	2.84	−992.29	60.04	0.00	0.00
ψ(4)	43.49	2.70	−995.43	60.03	−3.14	2.38
ψ(6)	53.02	3.25	−999.16	60.20	−6.87	3.56
ψ(3)	40.92	2.44	−1,044.70	61.14	−52.41	9.84
ψ(2)	38.64	2.32	−1,045.91	61.12	−53.62	9.94
ψ(1)	37.83	2.33	−1,049.73	61.24	−57.44	10.45
**Detection models (step 2)**						
*p*(4)	71.17	6.93	−979.88	59.33	0.00	0.00
*p*(2)	47.31	2.96	−985.54	58.15	−5.66	9.41
*p*(1)	45.55	2.84	−992.29	60.04	−12.40	10.88
*p*(3)	47.26	2.92	−993.16	60.21	−13.28	10.49

*Note that P_D_ represents the effective number of parameters rather than the true number of parameters. Leave-one-out expected log-predictive density (LOO), in addition to the difference in LOO between each model and the top-ranked model (Δ LOO), along with their standard errors (SE) are presented*.

All models were fitted using four parallel MCMC chains with 4,000 iterations, which included 2,000 iterations that were discarded as burn-in iterations for all candidate models; this always resulted in satisfactory convergence (Supplementary Figure 1), following the guidelines by Vehtari et al. (29). After the selection procedure, a prior sensitivity analysis was performed for the top-ranked model from step 2, by comparing results of the default prior specification with *Student t*(3, 0, 2.5) and *Student t*(3, 0, 10) priors for {α_*l*_, β_*l*_, σ_*f*_1__, σ_*f*_2__}; this analysis revealed posterior invariance under the considered prior specifications.

## Results

Table 2 presents the model selection process, which yielded a final model consisting of an occupancy process and detection process that will be detailed in the following subsections.

### Detectability

The detection model according to LOO (Table 2, *p*4) models temporal variation in wild boar's detectability for each observation month as a GP. Modeling the detection probability using biannual seasons (*p*2) results in a better fit compared to the intercept only model (*p*1). Using quarterly instead of biannual seasons, results in the lowest ranking detection model (*p*3). Note that the accuracy in Δ LOO, measured as the standard error of this metric, is relatively low for all detection models (Table 2, step 2). The posterior mean probability of detecting wild boar ranges between 0.0279 and 0.1106 regardless of the observation month. Despite low detectability in general, monthly differences can be observed (Supplementary Figures 2, 3).

### Occupancy

**Figure 2:**
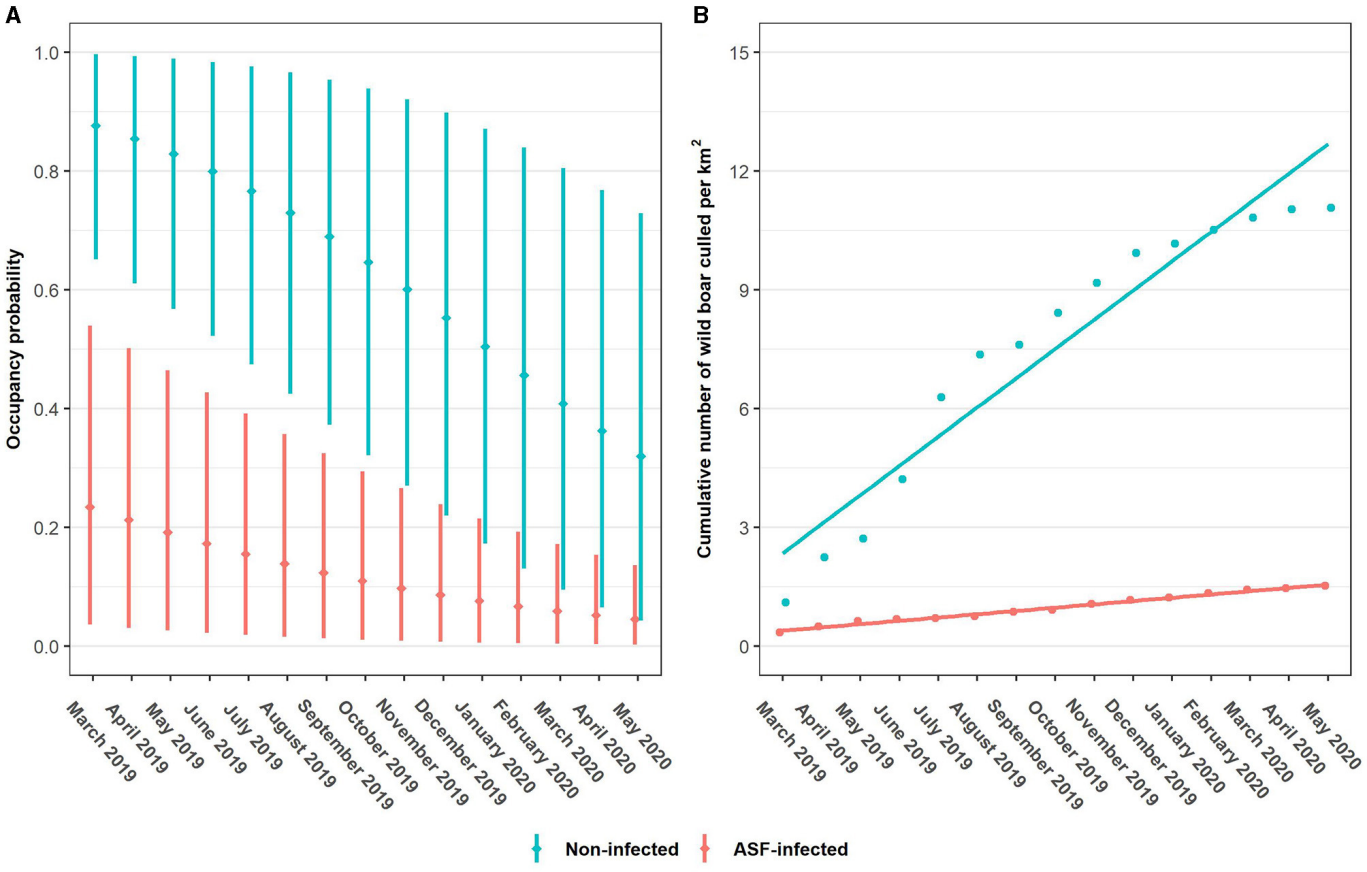
Monthly posterior mean occupancy estimates (dots) and 95% highest posterior density intervals (vertical lines) per ASF management zone **(A)**. Cumulative number of wild boar culled per km^2^ in function of the observation month. Trend lines derived from ordinary least squares regression estimation **(B)**.

**Figure 3:**
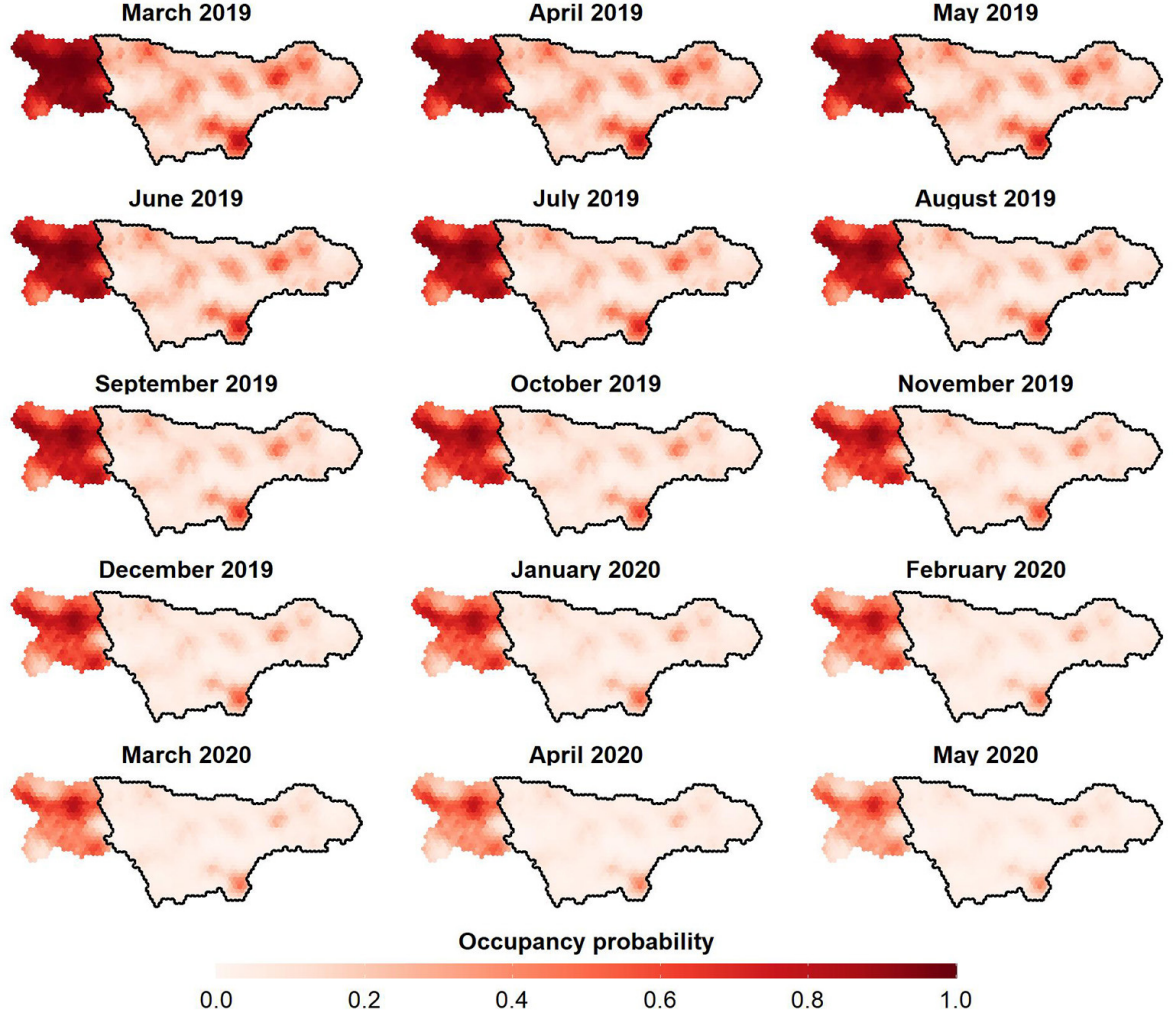
Posterior mean occupancy of wild boar in the ASF-infected (enclosed by the black line) and non-infected (non-enclosed) zone in Wallonia (Belgium). Panels ranging from March 2019 until May 2020.

All of the tested covariate combinations perform better than the intercept model (ψ1), with the multiplicative model of *ASF*_*i*_, *t* and *BL*_*t*_ (ψ5) outranking all other models. Similar to the detection models, not all Δ LOO values are very accurate. For the difference between the two top-ranking models the standard error exceeds |Δ LOO| (Table 2, step 1). According to the top-ranked occupancy model, ASF infection status has a strong effect on the occupancy of wild boar. Posterior mean odds ratios (OR) of wild boar occupancy are 17.71 (3.49–95.12) and 0.01 (0.00–0.08) for non-infected and ASF-infected zones, respectively. Moreover, the OR for season, 0.76 (0.65–0.88), reveals a highly probable overall decline in wild boar occupancy for observation month (*t*). Finally, the posterior distributions of ORs for both proportion of broad-leaved forest land cover class (*BL*_*t*_) and the ASF infection status—season interaction term (*ASF*_*i*_·*t*) have one enclosed by the 95% HPDI (Supplementary Table 4). Posterior mean occupancy and 95% HPDI at each observation month, averaged over the ASF management zones (Figure 2A), reveal an overall decline in occupancy, both in the ASF-infected and non-infected zone. Moreover, regression analyses using an ordinary least squares estimation of the cumulative number of wild boar culled per km^2^ result in positive trends for both zones (Figure 2B). Finally, prediction maps for the estimated occupancy from March 2019 until May 2020, are displayed in Figure 3 (see Supplementary Figures 4, 5 for 2.5th and 95th percentile maps).

### Occupancy Growth Rate

Posterior means of occupancy growth rates, i.e., λ_*it*_, are lower than one regardless of the site and season (Supplementary Figure 6). For λ_*Tot, i*_, total growth rates (in fact, extinction rates, due to their negative trend) in occupancy, posterior means range between 0.0865 and 0.7756 (0.9135 and 0.2244), while those for average monthly growth rates ${\bar{\lambda }}_{i}\;$lie between 0.8049 and 0.9757 (0.1951 and 0.1243). Finally, posterior mean and 95% HPDI for λ_*Tot, i*_ and ${\bar{\lambda }}_{i}\;$averaged over the ASF management zones (designated λ_*Tot, z*_ and ${\bar{\lambda }}_{z}$) are given in Table 3.

**Table 3:** Posterior mean and 95% highest posterior density values for the total growth rate (λ_*Tot, z*_) and average monthly growth rate (${\bar{\lambda }}_{z}$) per ASF management zone, obtained by averaging over all corresponding sites.

**Zone**	**Mean**	**2.5%**	**97.5%**	**Mean**	**2.5%**	**97.5%**
	${\bar{\lambda }}_{z}$			**λ_*****Tot*****, ***z***_**		
ASF-infected	0.8670	0.8083	0.9264	0.1772	0.0494	0.3432
Non-infected	0.9032	0.8105	0.9900	0.3848	0.0739	0.7946

## Discussion

To assess wild boar population trends throughout the recent ASF epidemic in Wallonia (Belgium), we have built a spatio-temporal MSO model using data from CTs. This was done according to a two-step approach, selecting the best detection covariates and subsequently occupancy covariates with respect to the LOO (28) statistics from a set of *a priori* defined models.

### Detectability

For all model comparisons (relative to the top-ranked detection model) the standard errors for Δ LOO values are smaller than two times |Δ LOO|, hence a certain degree of uncertainty as to which model provides the best fit to the data remains after our selection procedure (Table 2, step 2). Nevertheless, we believe that using a GP to model monthly temporal variation in wild boar detection probability is a sensible choice, given the ability of GPs to balance ecological realism with model flexibility (30).

When using CTs, detection probabilities are known to be affected by, among others, vegetation denseness, background surface temperature and weather conditions (31), all of which depend on the seasonal variation to some extent. Hence, a certain degree of seasonality in detection probabilities is not uncommon. Morelle et al. (17) report higher probabilities of detecting wild boar in summer months compared to fall (4.90E+04), winter (4.34E-03) and spring (4.90E+04). In this study, posterior mean detection probability for wild boar is low, although some additional heterogeneity attributed to the observation month was observed. In 2019, summer months display a somewhat higher probability of detecting wild boar as compared to winter months, yet there is insufficient evidence that a periodic trend exists. Instead, we suggest that the main effect at play is a density-dependent effect (32, 33), more specifically a decline in detection probability governed by a decreasing wild boar density. In addition, the intensive culling regime adopted throughout the ASF epidemic possibly led to an increased risk perception by wild boar, incentivizing them to restrict their movements and seek hiding places. Lower activity levels negatively relate with photographic rates (34). Similarly, low probabilities of detecting wild boar could reflect decreased movement.

### Occupancy

The top-ranked occupancy model consists of a multiplicative effect the ASF infection status (*ASF*_*i*_) of the zone, the observation month (*t*) and the proportion of deciduous forest land-use class (*BL*_*t*_), closely followed by a fully additive model of these covariates (Table 2, step 1). A large difference in wild boar occupancy was already present during the first month of the study period (March 2019), with posterior mean occupancies of 0.2352 and 0.8677 in infected and non-infected zones, respectively (Supplementary Table 5). Hence, ASF-governed mortality, which had already decimated the population in the infected zone at the beginning of the monitoring program (Supplementary Table 6), strongly affects wild boar occupancy [posterior mean OR($\beta_{ASF}^{\psi }$) of 0.01; Supplementary Table 4]. This is not surprising given that the zone was already infected with ASF for several months (i.e., since September 2019) before the study's onset. Although it is uncertain whether the low initial occupancy in the infected zone is driven by ASF alone, mortality rates approaching 100% have been reported (35). Interestingly, the inclusion of a HSGP that achieves a degree of spatial smoothing depending on ρ_*f*_2__, did not result in smoothly varying occupancies at the infected/non-infected boundary. Instead, the occupancy abruptly changes at this boundary, a trend that is seen throughout the entire study period (Figure 3). This pattern persists, even after omitting the information about ASF infection status (i.e., a potential driver of abrupt changes in occupancy, due to its binary encoding) from our model (results not shown). In that case, the variation in occupancy previously accounted for by $\beta_{ASF}^{\psi }$, remains explained by the spatial GP ($f_{2}^{\psi }$). Hence, we are confident that this absence of smooth occupancy patterns is not an artifact of our choice of covariates. Instead, we argue that fences placed at the infected/non-infected boundary (Figure 1) serve as an effective measure to stop the passage of wild boars, explaining the patterns observed in Figure 3. By impeding wild boar's movement, fences also prevent the inflow of ASFV throughout an epidemic. Hence, we regard fencing, as it was adopted in Wallonia (Belgium), as an essential element in the ASF-management strategy.

Despite the strong impact of ASF, the infected zone has quite some refugee sites that display higher occupancies compared to surrounding areas as of March 2019 (Figure 3). As the epidemic progressed, occupancy drops in most of these subareas, with only one patch in the South displaying markedly higher occupancy toward May 2020. Given the remoteness of this patch, it could be that wild boar in this area are shielded from ASF to some extent. A more likely explanation is that these refugee sites reflect the area's suitability for remaining wild boar in terms of habitat quality and food availability. Similarly, we argue that latent ecological preferences drive the heterogeneity in occupancy observed within the non-infected zone, where higher occupancies are observed in the central axis (horizontally) throughout the study period (Figure 3). Indeed, looking at the spatial random effect alone, both the Southern patch of the ASF-infected zone and central axis of the non-infected zone are associated with some of the highest values (Supplementary Figure 7). A number of potential ecological drivers of wild boar occupancy are observed and subsequently modeled; we have considered the proportion of broad-leaved tree land cover class, which is known to positively affect its occupancy (36–38), as a fixed effect in our final model. The 95% HPDI for the OR ($\beta_{BI}^{\psi }$) (Supplementary Table 4), which encompasses one, suggests an effect that needs further investigation in future studies.

Importantly, we obtain an overall declining trend [posterior mean OR ($\beta_{t}^{\psi }$) of 0.76; Supplementary Table 4] in wild boar occupancy for both ASF-infected an non-infected zones (Figure 2A). Interestingly, these declines are inversely related with the positive trends of the cumulative number of wild boar culled per km^2^ in function of the observation month (Figure 2B). Although hunting statistics are sensitive to search effort, these findings indicate that occupancy probabilities continue to drop in response to maintained culling efforts. In addition, ASF-induced mortality contributes to the occupancy decline seen in the ASF-infected zone, where its effect is strongest during the first months of our study period, when ASFV-positive wild boars are still found occasionally Supplementary Table 6. Finally, we find that occupancy declines have different rates between the zones, with a more moderate decline seen in the ASF-infected zone [posterior mean OR ($\beta_{ASF\cdot t}^{\psi }$) of 1.13; Supplementary Table 4]. Possibly, differences in hunting pressure could explain this variation in rates of occupancy decline. Although wild boars have been culled in both ASF-infected and non-infected zones, the latter was more densely populated throughout the entire study period, which likely reduces the search effort by hunters and leads to increased hunting success (Supplementary Table 7). In addition, we note that between the epidemic-onset and its peak (September 2019 – February 2019), an occupancy decline was likely much higher in the ASF-infected zone. Importantly, our model reveals that an effect of the *ASF*_*i*_·*t* interaction term is not highly probable when looking at 95% HPDI. Uncertainty about the existence of zone-specific occupancy rates, is also reflected in the small Δ LOO between a model with and one without the interaction term (Table 2, step 1).

### Occupancy Growth Rate

We will not discuss growth rates in depth, since they carry on the same messages as the occupancy probabilities discussed earlier, but see Supplementary Figure 6 for a graphical representation. However, it is worthwhile to briefly focus on total occupancy growth rates, as they provide a summary statistic for net change in occupancy. Posterior means of 0.1772 and 0.3848 (extinction rates of 0.9228 and 0.7152) for, respectively, ASF-infected and non-infected zones, confirm the strong decline in wild boar occupancy. In line with these results, Morelle et al. (17) report declines in wild boar abundance, obtained through fitting a REM (12) to CT data, of 83.8 ± 25.5% and 94.8 ± 6.4% in unmanaged and managed zones, respectively, one year after an ASF outbreak in the Białowieza Primeval Forest (Poland). Moreover, average monthly growth rates of 0.8670 and 0.9032 (Table 3) indicate that monitoring highly lethal diseases, such as ASF, which typically lead to rapid depletion of individuals, demands for shorter primary sampling periods.

### Limitations

The data used in this study do not cover the full ASF episode as it occurred in Wallonia (Belgium). As such, we are unable to report on the full course of the epidemic. Secondly, it has been reported by (27) that sample sizes smaller than 40 lead to insufficient power to detect declines in occupancy under most circumstances. Here, we deploy 69 cameras in the ASF-infected and only 23 cameras in the non-infected zone. Nonetheless, we were able to capture meaningful declines in occupancy for both zones throughout the study period. Importantly, both ASF management zones had sampling intensities higher than the best scenario (2% of sites sampled) considered by Banner et al. (27). From a modeler's perspective, we did not attempt a full spatio-temporal analysis. However, we believe that it is reasonable to assume that temporal dynamics in site-occupancy are spatially independent, given the relatively small surface area (ASF-infected: 162.826 km^2^, non-infected: 48.229 km^2^) of both zones in our study. Finally, we did not include structured and unstructured random effects for both the detection and occupancy process, due to unidentifiability.

## Conclusion

Based on our results, we conclude that ASF infection status was the main driver of wild boar occupancy at the beginning of the monitoring period, which led to higher occupancies in the ASF-infected zone compared to the non-infected zone. Moreover, we find that fences placed at the infected/non-infected boundary act as an effective barrier throughout the entire study period, resulting in abrupt changes in occupancy from one zone to the other. This suggests that wild boar's movement across this barrier is severely impeded, preventing inflow of the ASFV to the non-infected zone. Starting from March 2019, our model strongly supports an overall decline in occupancy until May 2020, presumably due to maintained culling efforts. Together, these results confirm (1) a declining trend in wild boar occupancy resulting from ASF (only in the infected zone) and ASF control measures implemented in Wallonia (Belgium), and (2) the potential of using a CT network to monitor wild boar population trends and impacts thereon during an ASF outbreak.

## Data Availability Statement

The datasets presented in this study can be found in online repositories. The names of the repository/repositories and accession number(s) can be found below: https://figshare.com/projects/African_Swine_Fever_Monitoring/115092.

## Ethics Statement

Ethical review and approval was not required for the animal study because the data used in this work were obtained through a non-invasive method (camera trapping), which does not disturb the natural behavior of animal.

## Author Contributions

MB: methodology, formal analysis, visualization and writing—original draft preparation. TN: methodology and validation. MF: methodology and visualization. AL, VD, and BM: resources. JC and NB: supervision. MB, TN, VD, AL, JC, and NB: writing—review & editing, conceptualization. Each author's contribution is described using the CRediT roles. All authors contributed to the article and approved the submitted version.

## Funding

MB and MF are PhD fellows, MB is funded by a BOF-mandate at Hasselt University, MF is funded by the Research Foundation – Flanders (FWO) (grant number 11E3220N). The camera trapping infrastructure was provided and funded by the Public Service of Wallonia. Services used in this work were provided by the VSC (Flemish Supercomputer Center), funded by the Research Foundation – Flanders (FWO) and the Flemish Government. Finally, the ecotope dataset, used in this work, is derived from the LifeWatch ecotope database, which is led by the Earth & Life Institute (UC Louvain) and funded by the Wallonia-Brussels Federation.

## Conflict of Interest

The authors declare that the research was conducted in the absence of any commercial or financial relationships that could be construed as a potential conflict of interest.

## Publisher's Note

All claims expressed in this article are solely those of the authors and do not necessarily represent those of their affiliated organizations, or those of the publisher, the editors and the reviewers. Any product that may be evaluated in this article, or claim that may be made by its manufacturer, is not guaranteed or endorsed by the publisher.
