# Supplementary material for: Managing African Swine Fever: Assessing the Potential of Camera Traps in Monitoring Wild Boar Occupancy Trends in Infected and Non-infected Zones, Using Spatio-Temporal Statistical Models
Source: Front Vet Sci. 2021 Oct 12;8:726117. doi: 10.3389/fvets.2021.726117 (PMC8546189; doi:10.3389/fvets.2021.726117)
Supplement: Supplementary file 1 [file Data_Sheet_1.docx]

Supplementary Material

# Supplementary Figures and Tables

## Supplementary Figures


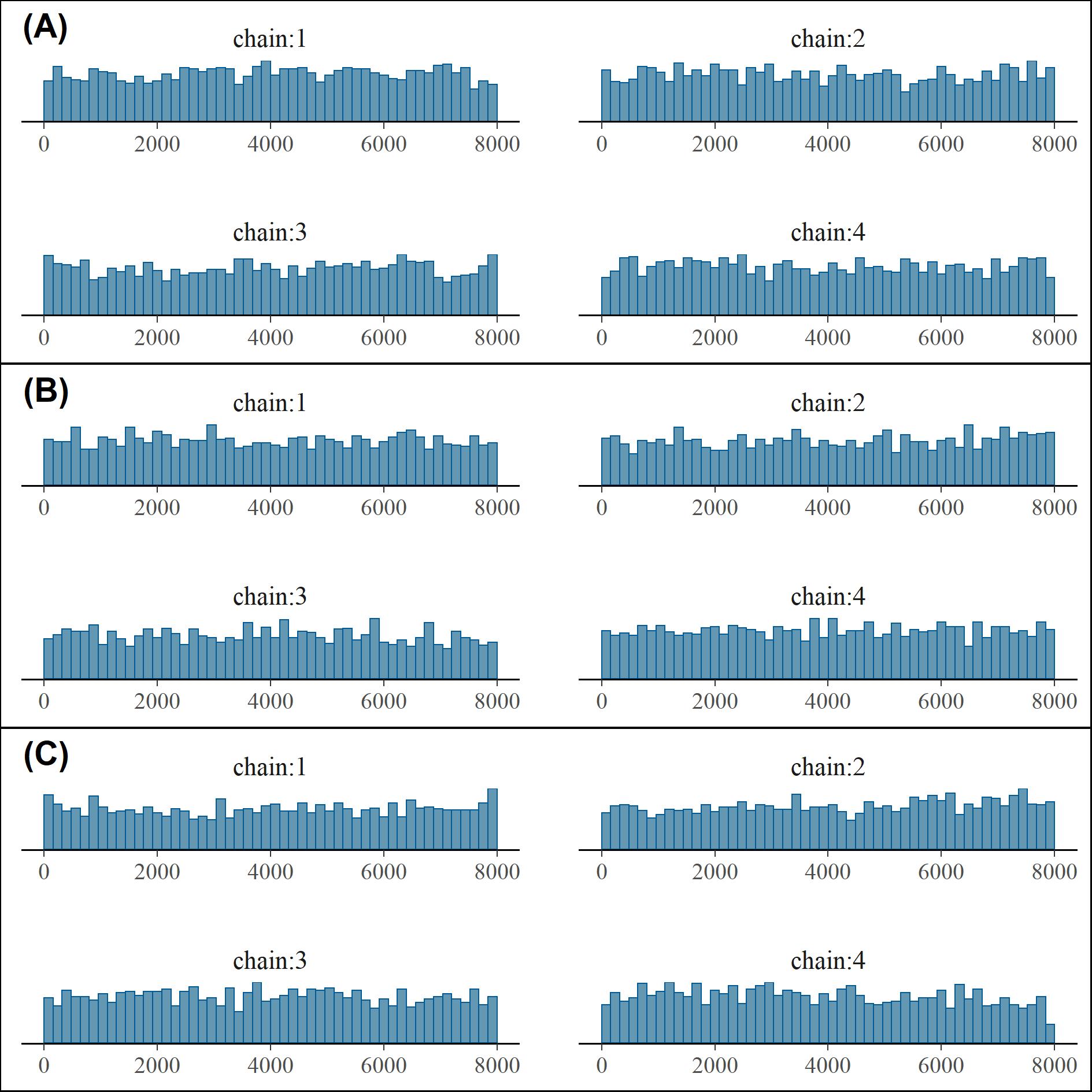


**Supplementary Figure 1.** Rank plots from posterior draws of the top-ranked occupancy model tracking the mixing of MCMC chains. Panels display rank plots for regression parameters${\{\alpha}_{l},\beta_{l}\}$ (**A**), parameters of the Gaussian process explaining temporal variation in detection probability $\left\{ \sigma_{f_{1}}, \rho_{f_{1}} \right\}$ (**B**) and spatial variation in occupancy $\left\{ \sigma_{f_{2}}, \rho_{f_{2}} \right\}$ (**C**). For all panels, only parameters with the lowest tail-ESS (effective sample size) are displayed.


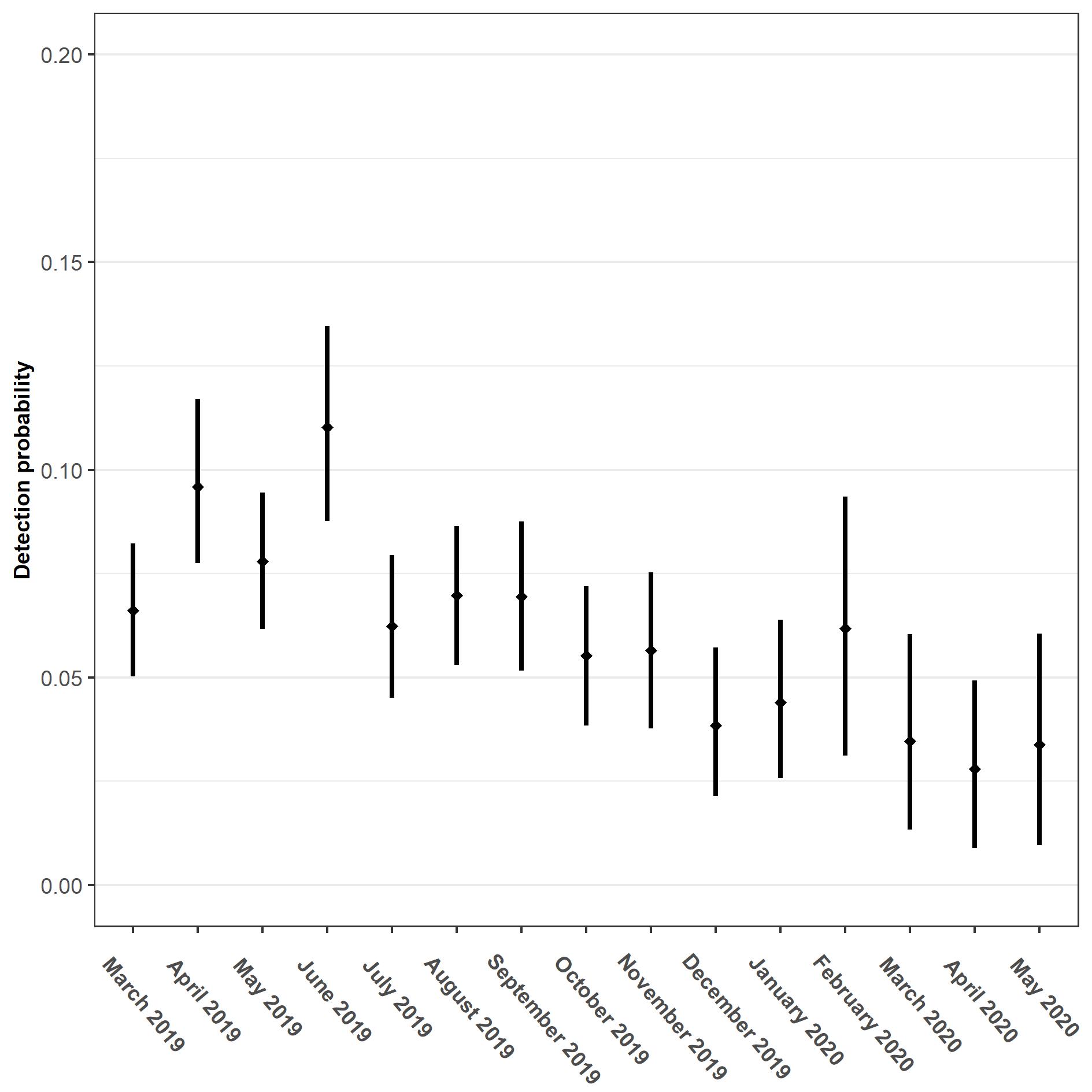


Supplementary Figure 2: Probability of detecting wild boar in response to the observation month. Posterior means (dots) and 95% highest posterior density intervals (vertical lines).


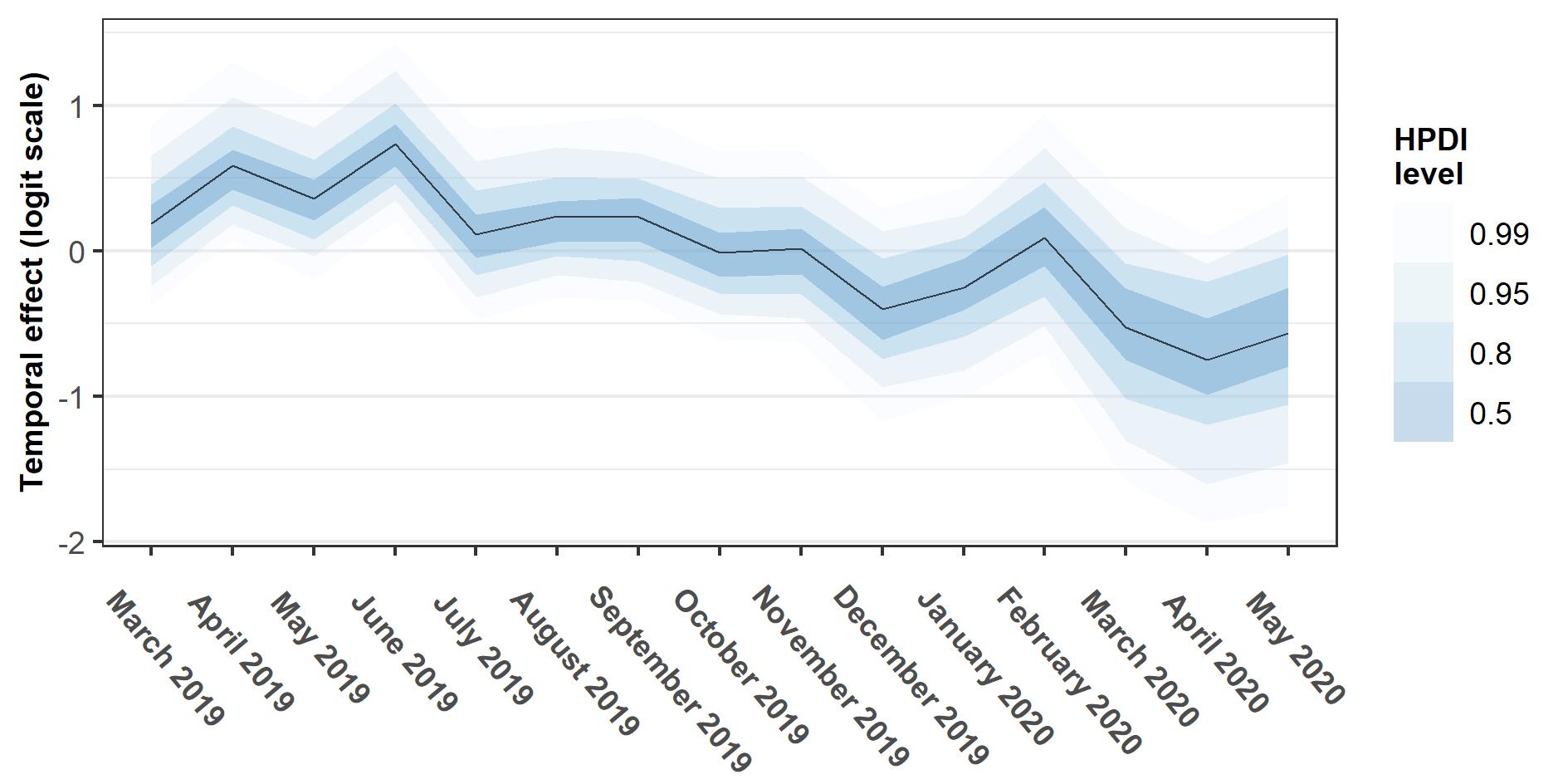


Supplementary Figure 3: Posterior mean and 50, 80, 90 and 95% highest posterior density intervals (HPDI) for the temporal effect (detection process) estimated by a Gaussian process. All values are presented on the logit scale.


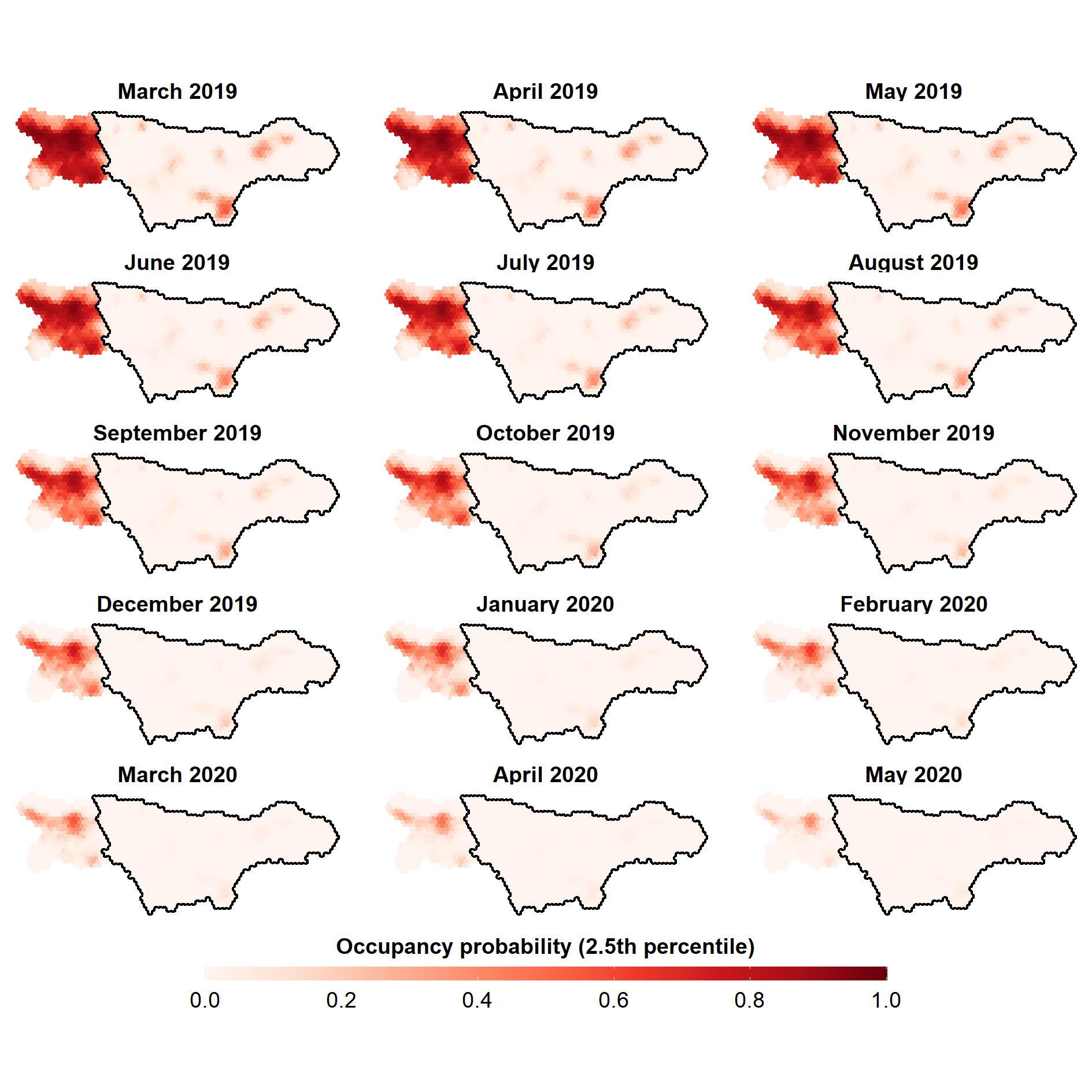


Supplementary Figure 4: 2.5^th^ Percentile of posterior occupancy of wild boar in the ASF-infected (enclosed by the black line) and non-infected (non-enclosed) zone in the Wallonia (Belgium). Panels ranging from March 2019 until May 2020.


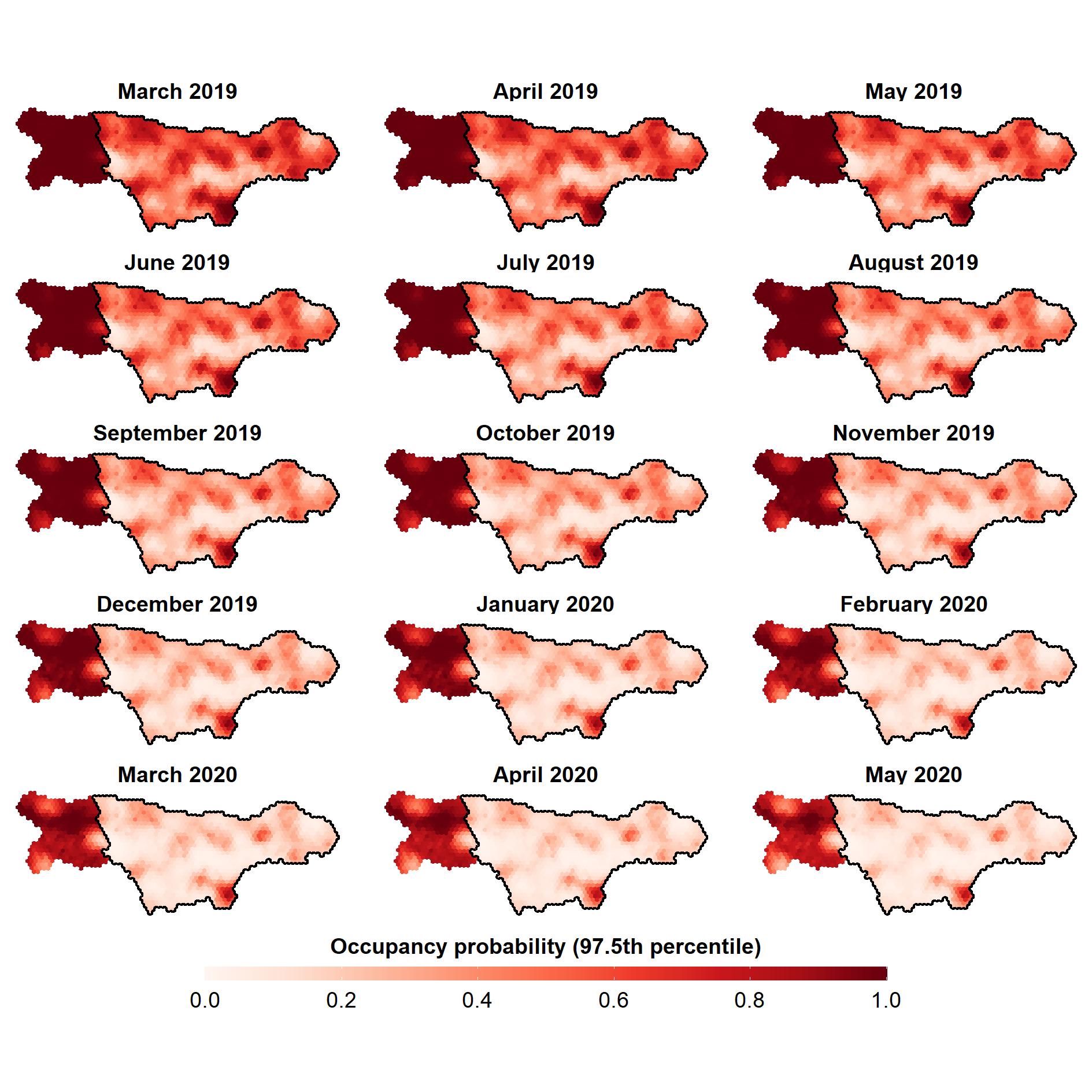


Supplementary Figure 5: 97.5^th^ Percentile of posterior occupancy of wild boar in the ASF-infected (enclosed by the black line) and non-infected (non-enclosed) zone in Wallonia (Belgium). Panels ranging from March 2019 until May 2020.


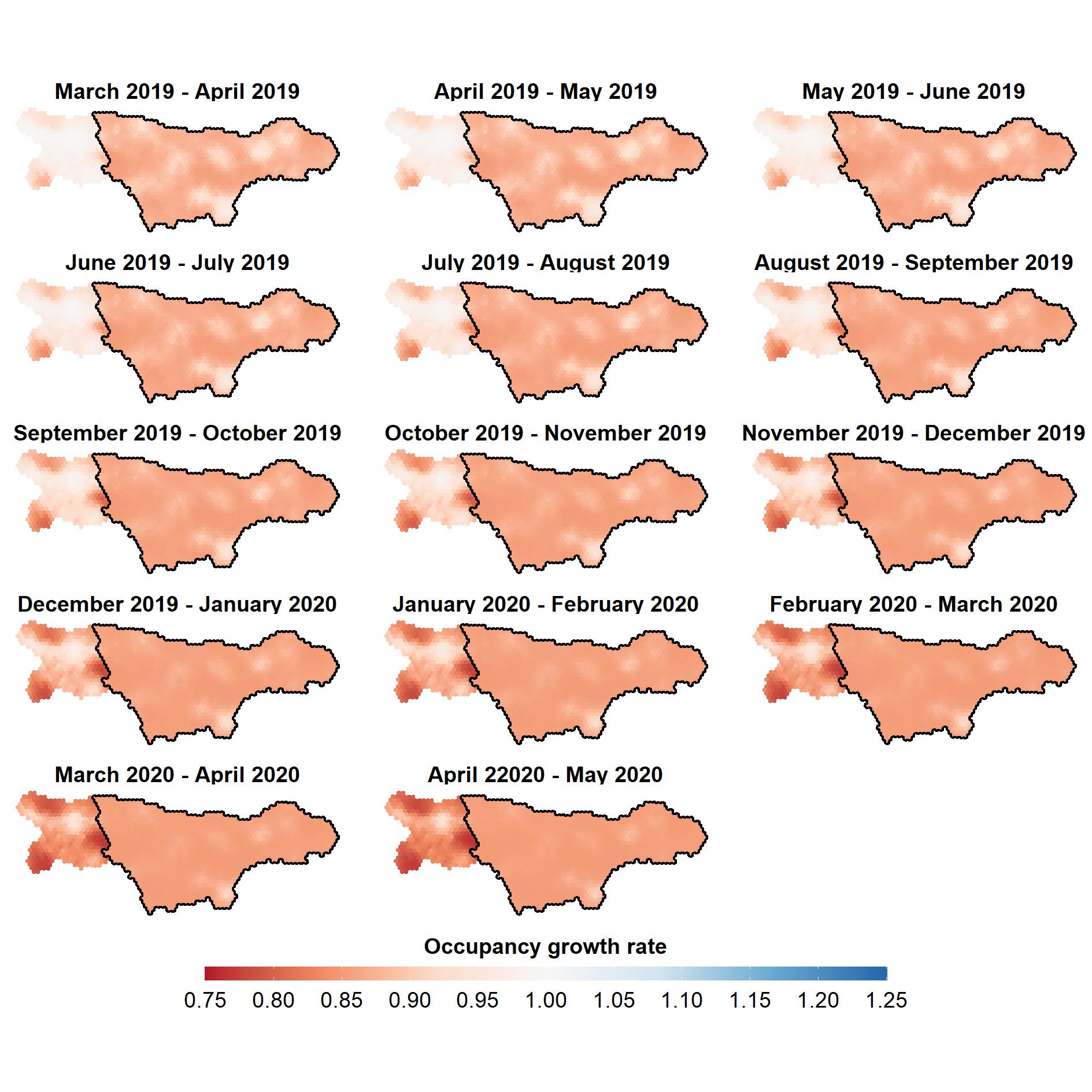


Supplementary Figure 6: Posterior mean occupancy growth rates of wild boar in the ASF-infected (enclosed by the black line) and non-infected (non-enclosed) zone in Wallonia (Belgium). Panels display growth rates derived from two consecutive months, ranging from March 2019 – April 2019 until April 2020 – May 2020.


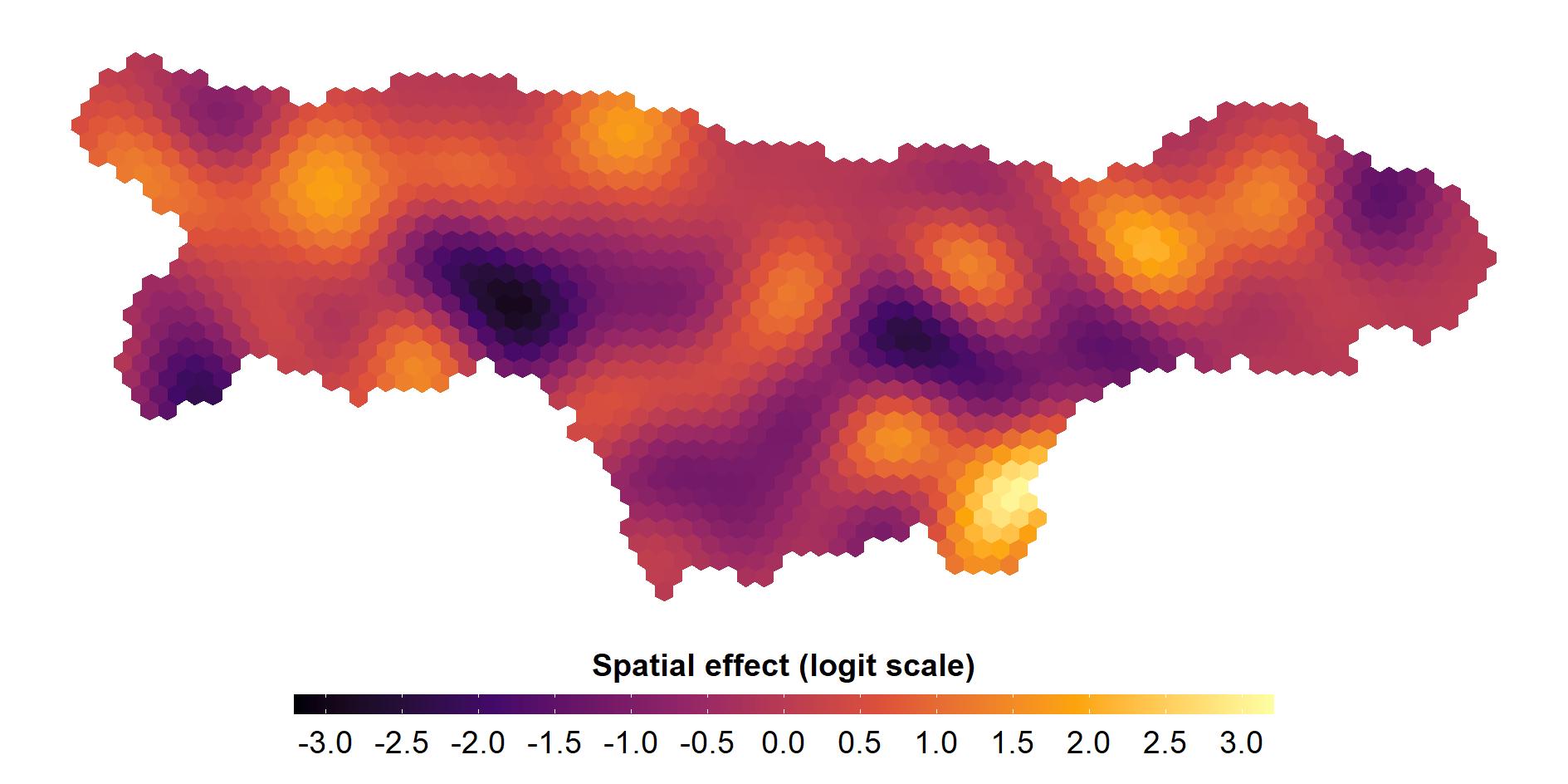


Supplementary Figure 7: Posterior mean of the spatial effect (occupancy process), estimated by a Hilbert-space Gaussian process. Mean values are presented on the logit scale.

## Supplementary Tables

Supplementary Table 1: Features and characteristics of the Snapshot Extra Black 12.0 | HD (Dörr) camera, as specified by the manufacturer.

| **Camera feature** | **Characteristics** |
| --- | --- |
| Motion sensor | Infrared |
| Trigger speed | 1/5 sec |
| Detection range | 14.6 m |
| Detection angle | 58° |

Supplementary Table 2: Sampling effort for each of the ASF management zones.

| **Zone** | **Total Grids** | **Number Sampled** | **Sampled (%)** |
| --- | --- | --- | --- |
| ASF-infected | 1135 | 69 | 6.08 |
| Non-infected | 348 | 23 | 6.61 |
| Excluded | 172 | 5 | 2.91 |

Supplementary Table 3: Predictions for *a priori* defined occupancy (step 1) and detection (step 2) models as defined in Table 1.

| **Model** | **Prediction** |
| --- | --- |
| Occupancy models (step 1) | |
| $\psi1$ | P($\psi1$): No selection. |
| $\psi2$ | P($\psi2$): Higher occupancy in non-infected sites. |
| $\psi3$ | P($\psi3$): Higher occupancy in non-infected sites with a high percentage of broad-leaved tree land cover class. |
| $\psi4$ | P($\psi4$): Similar as P($\psi2$), additionally an overall linear (declining) trend in occupancy exists. |
| $\psi5$ | P($\psi5$): Similar as P($\psi3$), but with different occupancy trends for ASF-infected and non-infected zones. |
| $\psi6$ | P($\psi6$): Similar as P($\psi2$), with the temporal trend in occupancy best captured by a flexible (non-linear) process. |
| Detection models (step 2) | |
| $p1$ | P($p1$): No selection. |
| $p2$ | P($p2$): Lower detectability during spring and summer due to denser vegetation cover. |
| $p3$ | P($p3$): Lowest detectability during summer due to denser vegetation cover, followed by spring and autumn. Highest detectability during winter. |
| $p4$ | P($p4$): Temporal trend in detectability best captured by a flexible (non-linear) process. |

Supplementary Table 4: Odds ratios for posterior means and 95% highest posterior density intervals, extracted from the top-ranked model. Only regression parameters are reported.

| **Parameter** | **Mean** | **2.5%** | **97.5%** |
| --- | --- | --- | --- |
| $\alpha^{p}$ | 17.71 | 3.49 | 95.12 |
| $\beta_{ASF}^{\psi}$ | 0.01 | 0.00 | 0.08 |
| $\beta_{BL}^{\psi}$ | 1.48 | 0.97 | 2.35 |
| $\beta_{t}^{\psi}$ | 0.76 | 0.65 | 0.88 |
| $\beta_{ASF\cdot t}^{\psi}$ | 1.13 | 0.97 | 1.32 |
| $\alpha^{\psi}$ | 0.06 | 0.04 | 0.08 |

Supplementary Table 5: Posterior Mean and 95% highest posterior density values for zone-averaged occupancy ($\psi_{t,z}$) estimates at observation month $t$.

| $\boldsymbol{t}$ | **Month** | **Mean** | **2.5%** | **97.5%** | **Mean** | **2.5%** | **97.5%** |
| --- | --- | --- | --- | --- | --- | --- | --- |
|  |  | ASF-infected | | | Non-infected | | |
| 2019 | | | | | | | |
| 1 | March | 0.2352 | 0.0366 | 0.5399 | 0.8677 | 0.6342 | 0.9958 |
| 2 | April | 0.2131 | 0.0312 | 0.5015 | 0.8453 | 0.5954 | 0.9922 |
| 3 | May | 0.1923 | 0.0265 | 0.4638 | 0.8197 | 0.5544 | 0.9875 |
| 4 | June | 0.1730 | 0.0224 | 0.4269 | 0.7907 | 0.5110 | 0.9814 |
| 5 | July | 0.1550 | 0.0188 | 0.3911 | 0.7584 | 0.4656 | 0.9741 |
| 6 | August | 0.1385 | 0.0156 | 0.3568 | 0.7227 | 0.4177 | 0.9642 |
| 7 | September | 0.1234 | 0.0129 | 0.3243 | 0.6838 | 0.3686 | 0.9522 |
| 8 | October | 0.1097 | 0.0106 | 0.2937 | 0.6421 | 0.3187 | 0.9372 |
| 9 | November | 0.0972 | 0.0087 | 0.2651 | 0.5980 | 0.2691 | 0.9191 |
| 10 | December | 0.0860 | 0.0070 | 0.2386 | 0.5523 | 0.2209 | 0.8972 |
| 2020 | | | | | | | |
| 11 | January | 0.0759 | 0.0056 | 0.2142 | 0.5057 | 0.1747 | 0.8711 |
| 12 | February | 0.0668 | 0.0045 | 0.1917 | 0.4592 | 0.1339 | 0.8418 |
| 13 | March | 0.0588 | 0.0035 | 0.1712 | 0.4135 | 0.0984 | 0.8091 |
| 14 | April | 0.0517 | 0.0027 | 0.1527 | 0.3696 | 0.0698 | 0.7747 |
| 15 | May | 0.0453 | 0.0022 | 0.1360 | 0.3281 | 0.0476 | 0.7388 |

Supplementary Table 6: Numbers of ASF virus-positive (ASFV+) wild boar found death per observation month $t$ in each of the ASF management zones throughout the study period. Note that the study’s onset is indicated by an horizontal line break and that all months prior to that are labelled using negative integers. Data presented in this table was adopted from Dellicour et al. (20).

| $\boldsymbol{t}$ | **Month** | **Number of ASFV+ wild boar found death (per km^2^)** | |
| --- | --- | --- | --- |
|  |  | ASF-infected | Non-infected |
| 2018 | | | |
| -6 | September | 52 (0.3194) | 0 (0.000) |
| -5 | October | 103 (0.6326) | 0 (0.000) |
| -4 | November | 71 (0.4361) | 0 (0.000) |
| -3 | December | 72 (0.4422) | 0 (0.000) |
| 2019 | | | |
| -2 | January | 184 (1.1301) | 9 (0.1866) |
| -1 | February | 111 (0.6818) | 17 (0.3526) |
| 1 | March | 24 (0.1474) | 0 (0.000) |
| 2 | April | 9 (0.0553) | 1 (0.0207) |
| 3 | May | 1 (0.0061) | 0 (0.000) |
| 4 | June | 2 (0.0123) | 0 (0.000) |
| 5 | July | 0 (0.000) | 0 (0.000) |
| 6 | August | 1 (0.0061) | 0 (0.000) |

Supplementary Table 7: Numbers of wild boar culled per observation month $t$ in each of the ASF management zones throughout the study period.

| $\boldsymbol{t}$ | **Month** | **Number of wild boar culled (per km^2^)** | |
| --- | --- | --- | --- |
|  |  | ASF-infected | Non-infected |
| 2019 | | | |
| 1 | March | 56 (0.3440) | 53 (1.0991) |
| 2 | April | 23 (0.1413) | 55 (1.1406) |
| 3 | May | 23 (0.1413) | 23 (0.4770) |
| 4 | June | 9 (0.0553) | 72 (1.4932) |
| 5 | July | 3 (0.0184) | 100 (2.0738) |
| 6 | August | 9 (0.0553) | 52 (1.0784) |
| 7 | September | 17 (0.1044) | 12 (0.2489) |
| 8 | October | 8 (0.0491) | 39 (0.8088) |
| 9 | November | 24 (0.1474) | 36 (0.7466) |
| 10 | December | 18 (0.1106) | 37 (0.7673) |
| 2020 | | | |
| 11 | January | 10 (0.0614) | 11 (0.2281) |
| 12 | February | 18 (0.1106) | 17 (0.3526) |
| 13 | March | 14 (0.0860) | 15 (0.3111) |
| 14 | April | 5 (0.0307) | 10 (0.2074) |
| 15 | May | 11 (0.0676) | 2 (0.0415) |
